# Supplementary material for: Dominant remodelling of cattle rumen microbiome by Schedonorus arundinaceus (tall fescue) KY-31 carrying a fungal endophyte
Source: Access Microbiol. 2022 Feb 24;4(2):000322. doi: 10.1099/acmi.0.000322 (PMC8941964; doi:10.1099/acmi.0.000322)
Supplement: Supplementary material 1 [file acmi-4-0322-s001.pdf]

Revised Manuscript ACMI-D-21-00194, December 8, 2021

Dominant Remodeling of Cattle Rumen Microbiome by *Schedonorus arundinaceus* (Tall Fescue) KY-31 Carrying a Fungal Endophyte

Supplementary Materials

Bela Haifa Khairunisa[1], Dwi Susanti[2#a], Usha Loganathan[2], Christopher D. Teutsch[3#b], Brian T. Campbell[3#c], David Fiske[4†], Carol A. Wilkinson[3], Frank O. Aylward[5], Biswarup Mukhopadhyay[2]

[1]Genetics, Bioinformatics, and Computational Biology, Virginia Tech, Blacksburg, VA 24061, USA

[2]Department of Biochemistry, Virginia Tech, Blacksburg, VA 24061, USA

[3]Southern Piedmont Agricultural Research and Extension Center, Virginia Tech, Blackstone, VA 23824, USA

[4]Shennandoah Valley Agricultural Research and Extension Center, Virginia Tech, Raphine, VA, 24472, USA

[5]Department of Biological Sciences, Virginia Tech, Blacksburg, VA 24061, USA.

[#a]Current address: Elanco Animal Health, Greenfield, IN, USA.

[#b]Current address: University of Kentucky Research and Education Center, Princeton, KY, USA.

[#c]Current address: Archer Daniels Midland Company, Decatur, IL, USA.

Keywords: cattle, pasture, grazing, rumen microbiome, remodeling, tall fescue, KY-31, MaxQ, fescue toxicosis, ergovaline, degradation

Correspondence should be addressed to Biswarup Mukhopadhyay; biswarup@vt.edu

† Deceased

Supplementary Materials

**Method S1.**	Rumen sample collection
**Method S2.**	DNA extraction
**Fig. S1.**	Custom made sample collection system
**Fig. S2.**	Sample rarefaction of tall fescue rumen microbiome
**Table S1**.	Rumen samples included and excluded from the analysis
**Table S2.**	Core microbiome - OTUs present with 95% Prevalence
**Table S3**.	Microbiota shift due to grazing transitions
**Table S4.**	Microbiota shift in specific rumen fraction (solid and liquid) due to grazing transitions
**Table S5.**	Differentially abundant prokaryotic OTUs in the rumen of steers susceptible to fescue toxicosis with respect to that of tolerant steers
**Table S6.**	Phylum-level microbiota comparison between steers graze on two tall fescue cultivars and other diets

Method S1. Rumen sample collection

Samples of rumen contents (fluid and fiber mat) were collected at intervals as described in the Results, using a custom-made sampling device consisting of a motorized vacuum pump that was connected to a vacuum chamber (Fig. S1). The chamber contained a fixed holder (SCH, Fig. S1) for a sample container (SC, Fig. S1), a beaker, and a slot at the top through which a removable collection tube could be inserted. A separate collection tube was used for each steer in order to prevent cross contamination. Each collection tube was made of a clear vinyl tube (VT, Fig. S1; Watts RVSP 1 5/8 in O.D. x 1 ¼ in I.D., Cat. No: 048643-025691, Watts Water Technologies, MA) connected to a piece of PVC pipe (PP, Fig. S1; 1 in diameter PVC 1120 pipe, TrueFit® system 04010, Charlotte Pipe and Foundry, Charlotte, NC) on both ends. One end was designed to fit into the vacuum chamber (VC, Fig. S1) of the sampling device and the other end for inserting into the rumen (Canula, Fig. S1). These collection tubes and sample containers were washed prior to using with Tergazyme, a detergent (Alconox Inc, White Plains, NY), and 2% phosphoric acid to remove microbial and nucleic acid contaminations. Additional details appear in the main manuscript.

Method S2. DNA extraction

DNA was purified from each rumen sample via bead beating and using a modified version of a protocol involving extraction with mixtures phenol, chloroform, and isoamyl alcohol (25:24:1, v/v/v) (PCI) and chloroform and isoamyl alcohol (24:1, v/v) (CI), followed by isopropanol precipitation and ethanol wash (24). In brief, 0.2 ml of a liquid fraction was mixed with 0.4 ml 1.5-fold concentrated extraction buffer or 0.4 gram of solid materials was mixed with 1 ml of extraction buffer in a 2 ml microcentrifuge tube; the extraction buffer contained 100 mM Tris-HCl, 100 mM EDTA, 1% CTAB, and 500 mM NaCl. Microbial cells that were attached to solid material were

released via vortexing with 1 mm zirconia/silica beads (Bio Spec Inc., Bartlesville, OK), that have been washed with 2% phosphoric acid prior to the use, using a VWR Standard Heavy-Duty Vortex Mixer at 2,500 rpm (VWR International, LCC, Radnor, PA). The microbial cells were then lysed via vortexing with 0.1 mm acid washed silica beads (Bio Spec Inc.), followed by the addition of lysozyme and RNase (Sigma-Aldrich, St. Louis, MO) to final concentrations of 0.37 mg/ml and 0.5 mg/ml, respectively. The mixture was incubated at 37°C for 30 minutes and vortexed for 1 min. Following this step, sodium dodecyl sulfate (SDS) and proteinase K (New England BioLabs, Ipswich, MA) were added to final concentrations of 2% and 0.0735 mg/ml, respectively, and the mixture was incubated at 60°C for one hour. The DNA from this mixture was purified via extraction with PCI and CI and precipitation with isopropanol and sodium acetate, washed with 75% ethanol in water, dried under air, and dissolved in 200 µl of nuclease-free water. Due to a high level of humic acid carry over in DNA extracted from solid samples, a second precipitation with polyethylene glycol (PEG 8000) and NaCl at final concentrations of 6% and 0.6 M, respectively, was performed at 4°C. After overnight incubation with PEG, DNA was recovered by centrifugation at 16,000 x g for 20 minutes at 4°C followed by two washes with 75% ethanol. The DNA pellet from this step was air dried and dissolved in 200 µl nuclease-free water. All DNA solutions were stored in a -20°C freezer until further use. Quantification and quality assessment of DNA samples were done via both agarose gel electrophoresis and nanodrop measurement (NanoDrop Lite UV-Vis Spectrophotometer, ThermoFisher Scientific, Waltham, MA).

References are listed in the main manuscript.

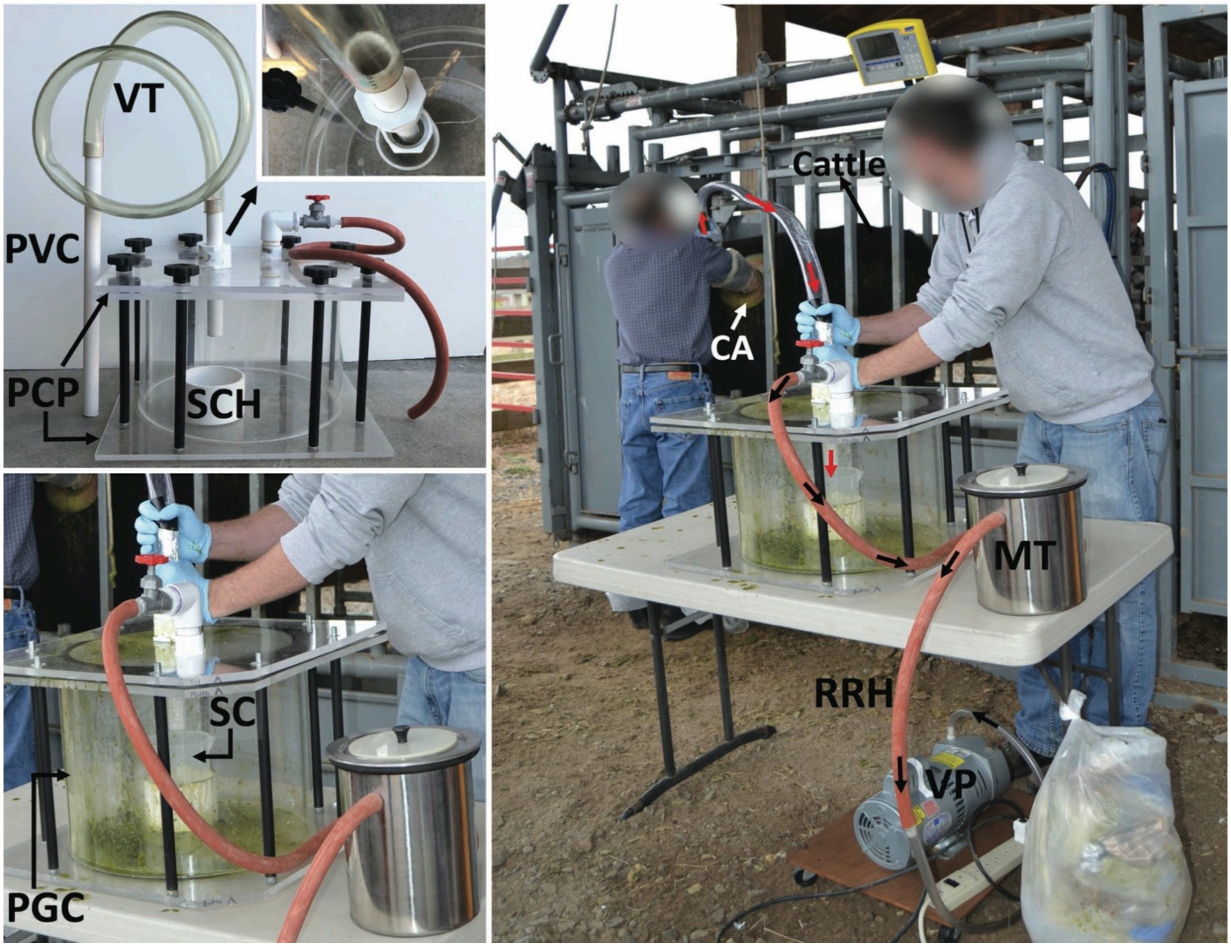

Figure S1. Custom made rumen sample collection system. Cattle, Hereford-Angus cross steer; CA: Cannula, 4-ich cannula (Bar Diamond Inc. Parma, ID); PVC: PVC pipe (acid washed); VT: vinyl tube (acid washed); SC: sample container (acid washed plastic beaker); SCH: sample container holder; PGC: plexiglass cylinder (diameter, 18 in; wall thickness, ¼ in); PGP: plexiglass plates (thickness, ½ in); RRH: red rubber hose; MT: Labconco dry ice moisture trap (catalog number 7538000, Thermo Fisher Scientific, Waltham, MA); VP: vacuum pump; red arrow: flow direction for sample; black arrow: direction for vacuum.

89

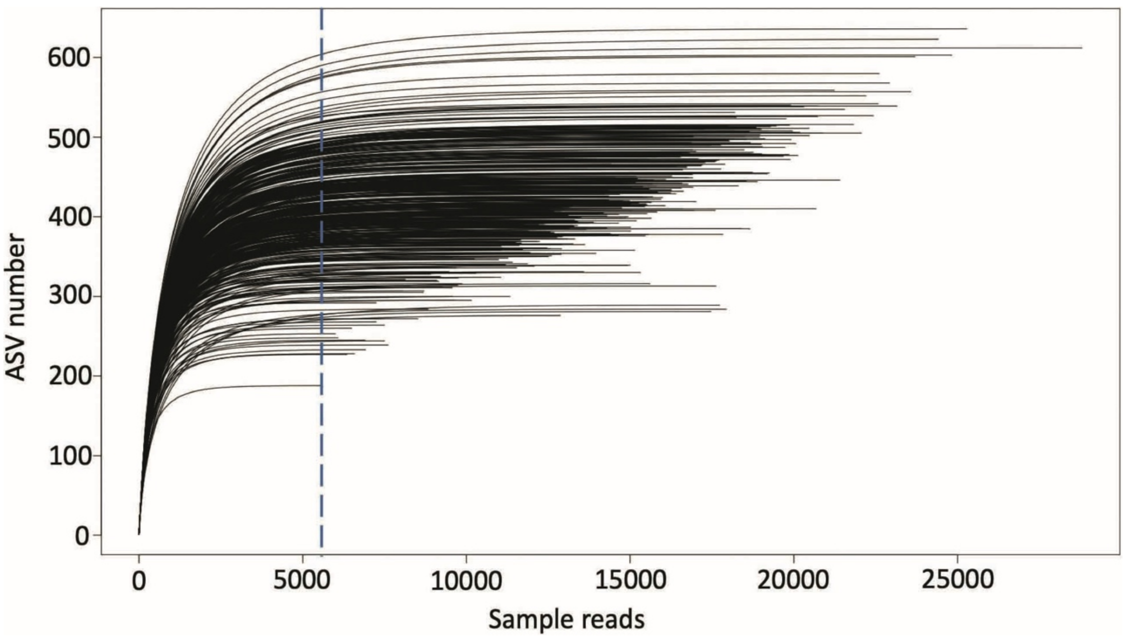

Figure S2. Sample rarefaction of tall fescue rumen microbiome. Rarefaction of rumen samples grazing two tall fescue cultivars. All of the samples were rarefied into equal sequencing depth of 5598 reads and then used for alpha diversity analysis.

Table S1. Rumen samples included and excluded from the analysis

Sample ID	Read count	Status
2Solid_EIII1	0	Excluded
4Solid_CII1	0	
2Solid_CI1	1	
2Solid_BIII1	112	
2Solid_DIII2	723	
4Liquid_GII2	997	
4Solid_FIII3	1636	
2Liquid_HI1	2194	
4Liquid_CIII1	3031	
4Solid_FII1	3712	
4Solid_AII2	3773	
4Solid_AII3	5197	
4Solid_DI2	5598	Included
2Liquid_CIII3	5995	
6Liquid_CI3	6075	
4Solid_FIII1	6348	
2Liquid_DI3	6491	
2Liquid_GII3	6579	
6Liquid_GII3	6902	
4Solid_DI3	6917	
2Liquid_FI3	7238	
6Liquid_BI1	7240	
4Solid_FIII2	7458	
4Solid_AII1	7488	
2Liquid_GIII1	7495	
4Solid_DI1	7608	
2Liquid_FI1	8070	
2Solid_EI3	8132	
2Liquid_BII1	8304	
4Liquid_AI3	8510	
2Solid_BII1	8687	
2Liquid_EI3	8697	
4Solid_HI1	8830	
6Liquid_CII3	8904	
2Liquid_AIII1	9016	
4Liquid_CI3	9092	
6Liquid_EI2	9157	
6Liquid_CII1	9205	
6Liquid_HI3	9267	
6Solid_CI3	9462	
2Liquid_CIII1	9560	
2Liquid_DII3	9568	
4Solid_HII3	9587	
4Liquid_AIII1	9661	
2Liquid_AII3	9700	
4Solid_EIII3	9731	
2Liquid_DII2	9739	
4Solid_GIII1	9871	

continued/-

Table S1. Rumen samples included and excluded from the analysis

continued from previous page

Sample ID	Read count	Status
4Liquid_EIII1	10137	Included
4Solid_CI3	10146	
2Liquid_BII3	10190	
4Solid_EIII1	10203	
2Solid_HII2	10242	
2Liquid_DI1	10247	
2Liquid_EIII3	10292	
2Liquid_EIII2	10408	
2Liquid_HII2	10449	
2Liquid_AII1	10686	
2Liquid_HI2	10767	
2Liquid_GII2	10838	
2Solid_EII3	10838	
2Liquid_DI2	10972	
4Solid_GIII3	10980	
6Liquid_BII2	10981	
2Liquid_EI2	11049	
2Liquid_CII1	11050	
4Solid_BII2	11050	
2Liquid_BIII3	11051	
2Solid_EIII2	11096	
2Liquid_EII3	11108	
6Solid_EIII3	11114	
4Liquid_BIII2	11117	
2Liquid_EII2	11141	
6Solid_AI1	11154	
2Solid_FII3	11168	
2Liquid_DIII2	11204	
4Solid_EI1	11268	
6Liquid_EII1	11313	
4Solid_AI2	11325	
2Liquid_FII2	11330	
2Liquid_AII2	11355	
2Liquid_BII2	11391	
4Solid_HII2	11399	
2Liquid_HI3	11406	
2Liquid_HII1	11489	
6Solid_BI2	11493	
6Liquid_DIII2	11503	
2Liquid_HIII1	11506	
4Solid_FI3	11531	
2Liquid_EI1	11572	
2Solid_HIII3	11617	
2Liquid_BI1	11647	
4Liquid_FIII1	11657	
4Liquid_FIII3	11667	
2Liquid_BI3	11701	
2Liquid_GI2	11717	

continued/-

Table S1. Rumen samples included and excluded from the analysis

continued from previous page

Sample ID	Read count	Status
4Solid_HIII2	12929	Included
4Liquid_CIII2	12935	
4Liquid_FII3	12937	
4Liquid_BI2	12981	
6Solid_AIII2	13092	
4Solid_EII2	13104	
2Liquid_CI3	13108	
2Liquid_CI1	13143	
6Liquid_BI3	13145	
2Liquid_FI2	13166	
2Liquid_CI2	13180	
6Liquid_DI3	13225	
2Solid_AI2	13244	
2Solid_GII2	13245	
4Solid_CII2	13249	
4Solid_FI1	13267	
4Liquid_BIII3	13282	
2Solid_GI2	13291	
4Liquid_EII2	13295	
4Solid_FI2	13310	
4Liquid_HI1	13353	
6Liquid_AII3	13372	
2Liquid_GII1	13394	
6Liquid_FI1	13394	
2Liquid_GI3	13398	
4Liquid_DII2	13403	
4Liquid_EII3	13412	
6Liquid_HII3	13412	
6Solid_CI1	13488	
4Liquid_EI3	13504	
2Liquid_CII2	13520	
4Solid_AIII1	13577	
4Solid_CIII2	13617	
4Solid_EIII2	13643	
6Liquid_DIII1	13675	
2Liquid_AI1	13694	
6Liquid_AIII1	13700	
4Solid_CI2	13704	
4Liquid_GI1	13753	
2Liquid_AIII2	13775	
6Liquid_EIII1	13782	
2Liquid_GIII2	13809	
4Solid_CIII1	13873	
4Liquid_FI1	13882	
2Solid_EI1	13916	
2Solid_AIII1	13948	
4Solid_AI1	13953	
4Liquid_BI2	12981	

continued/-

Table S1. Rumen samples included and excluded from the analysis

continued from previous page

Sample ID	Read count	Status
6Solid_AIII2	13092	**Included**
4Solid_EII2	13104	
2Liquid_CI3	13108	
2Liquid_CI1	13143	
6Liquid_BI3	13145	
2Liquid_FI2	13166	
2Liquid_CI2	13180	
6Liquid_DI3	13225	
2Solid_AI2	13244	
2Solid_GII2	13245	
4Solid_CII2	13249	
4Solid_FI1	13267	
4Liquid_BIII3	13282	
2Solid_GI2	13291	
4Liquid_EII2	13295	
4Solid_FI2	13310	
4Liquid_HI1	13353	
6Liquid_AII3	13372	
2Liquid_GII1	13394	
6Liquid_FI1	13394	
2Liquid_GI3	13398	
4Liquid_DII2	13403	
4Liquid_EII3	13412	
6Liquid_HII3	13412	
6Solid_CI1	13488	
4Liquid_EI3	13504	
2Liquid_CII2	13520	
4Solid_AIII1	13577	
4Solid_CIII2	13617	
4Solid_EIII2	13643	
6Liquid_DIII1	13675	
2Liquid_AI1	13694	
6Liquid_AIII1	13700	
4Solid_CI2	13704	
4Liquid_GI1	13753	
2Liquid_AIII2	13775	
6Liquid_EIII1	13782	
2Liquid_GIII2	13809	
4Solid_CIII1	13873	
4Liquid_FI1	13882	
2Solid_EI1	13916	
2Solid_AIII1	13948	
4Solid_AI1	13953	
6Liquid_BIII1	13981	
4Solid_HIII3	14012	
4Liquid_DIII2	14045	
4Liquid_BIII1	14061	
6Solid_HII2	14179	

continued/-

Table S1. Rumen samples included and excluded from the analysis

continued from previous page

Sample ID	Read count	Status
4Liquid_HI3	14202	Included
6Solid_EIII1	14244	
2Solid_DII1	14248	
2Solid_GI1	14283	
6Liquid_FI2	14285	
4Liquid_EII1	14286	
4Liquid_HII1	14305	
2Liquid_HIII3	14314	
2Liquid_FII3	14338	
6Liquid_HII2	14339	
4Solid_CII3	14340	
2Liquid_GIII3	14395	
2Liquid_AI2	14412	
6Liquid_DII1	14417	
6Liquid_HII1	14435	
2Liquid_CII3	14502	
4Liquid_FIII2	14543	
2Liquid_DIII1	14594	
2Solid_EII1	14625	
4Solid_DIII3	14637	
4Solid_GI3	14643	
6Solid_HIII3	14646	
4Liquid_EI2	14666	
6Solid_FIII3	14677	
4Liquid_CII1	14695	
4Solid_BIII3	14713	
4Liquid_HII2	14735	
4Solid_DIII2	14745	
6Solid_AII2	14746	
6Liquid_AIII2	14748	
6Liquid_EI3	14760	
6Solid_CIII3	14795	
4Liquid_DII3	14834	
6Solid_AII1	14859	
4Solid_DII1	14870	
6Liquid_GIII3	14914	
4Solid_BI2	14936	
2Solid_FI2	14949	
4Liquid_DI3	14980	
6Solid_HII1	14982	
4Solid_BI1	14984	
6Solid_CI2	14989	
2Liquid_AIII3	14998	
4Liquid_CII2	15003	
4Solid_GI2	15012	
2Solid_CI2	15019	
6Solid_GIII1	15032	
4Liquid_FI3	15047	

continued/-

Table S1. Rumen samples included and excluded from the analysis

continued from previous page

Sample ID	Read count	Status
6Liquid_EIII3	15049	Included
4Solid_GI1	15057	
6Solid_AI3	15114	
6Liquid_GII1	15140	
4Liquid_FI2	15154	
2Liquid_FIII3	15183	
4Liquid_AIII2	15190	
4Liquid_HIII3	15228	
2Solid_EI2	15264	
4Liquid_AII1	15313	
4Liquid_GI3	15377	
4Solid_BII3	15382	
4Liquid_HIII1	15389	
2Solid_EII2	15453	
4Solid_AI3	15462	
2Liquid_HIII2	15464	
6Solid_BII1	15469	
6Liquid_FIII3	15488	
2Solid_GI3	15510	
6Solid_HII3	15516	
4Solid_HII1	15518	
4Solid_AIII2	15555	
6Liquid_FI3	15578	
4Liquid_BII1	15583	
2Solid_HIII2	15593	
2Solid_GII1	15636	
4Solid_DIII1	15642	
4Solid_AIII3	15658	
4Liquid_CIII3	15671	
4Liquid_FII2	15671	
6Liquid_HIII3	15683	
6Liquid_EIII2	15684	
4Liquid_EIII2	15716	
2Solid_BIII2	15717	
6Solid_EII3	15750	
4Solid_HIII1	15809	
2Solid_DI1	15823	
6Liquid_HIII1	15837	
4Liquid_FII1	15846	
4Liquid_BII2	15881	
4Liquid_AIII3	15932	
4Solid_FII2	15999	
6Liquid_AII1	16033	
2Solid_HI3	16080	
2Liquid_DIII3	16081	
4Solid_EI2	16233	
6Solid_DII2	16254	
2Solid_DII2	16256	

continued/-

Table S1. Rumen samples included and excluded from the analysis

continued from previous page

Sample ID	Read count	Status
6Liquid_DII3	16268	Included
6Liquid_AIII3	16273	
6Solid_EIII2	16336	
4Liquid_HII3	16397	
2Solid_BI1	16398	
6Solid_FI3	16399	
4Liquid_EI1	16411	
6Liquid_CI2	16510	
4Liquid_GII3	16524	
4Solid_GII2	16544	
6Liquid_CI1	16558	
4Liquid_GIII3	16562	
2Solid_FI3	16577	
6Liquid_DIII3	16588	
4Liquid_DI2	16595	
4Solid_HI3	16597	
6Solid_BI1	16599	
6Liquid_EII3	16618	
6Solid_FII1	16627	
2Solid_BI2	16684	
4Solid_BII1	16721	
6Liquid_BII1	16734	
4Liquid_DI1	16737	
6Solid_GII1	16737	
6Liquid_DII2	16758	
2Solid_HI1	16771	
4Liquid_CI2	16810	
2Solid_HIII1	16853	
6Liquid_AI1	16870	
6Solid_BIII1	16903	
2Solid_GIII2	16904	
4Liquid_GIII1	16908	
2Solid_DI2	16914	
2Solid_CIII2	16918	
6Liquid_EI1	16928	
2Solid_CII2	16944	
6Solid_CII2	17005	
2Solid_CI3	17021	
2Solid_AIII3	17091	
4Solid_GII1	17162	
2Solid_HI2	17203	
6Solid_EII1	17256	
6Solid_BIII2	17280	
6Solid_DII1	17284	
6Solid_HIII1	17411	
6Liquid_GI1	17464	
6Solid_EII2	17484	
2Solid_CIII1	17515	

continued/-

Table S1. Rumen samples included and excluded from the analysis

continued from previous page

Sample ID	Read count	Status
4Liquid_BII3	17589	**Included**
4Liquid_AII3	17602	
4Liquid_AI2	17604	
6Solid_GI1	17624	
4Liquid_DIII3	17632	
6Liquid_GI2	17727	
2Solid_AII1	17729	
4Liquid_CI1	17759	
6Liquid_FIII2	17768	
6Liquid_GIII1	17827	
6Liquid_HIII2	17897	
6Liquid_GI3	17931	
2Solid_BI3	17973	
4Liquid_CII3	18000	
6Liquid_BII3	18041	
6Solid_DIII3	18109	
6Solid_AII3	18169	
2Solid_FIII1	18203	
6Liquid_BIII3	18206	
6Liquid_AI2	18208	
2Solid_BII3	18241	
2Solid_CIII3	18302	
6Solid_BI3	18382	
6Liquid_GII2	18401	
4Liquid_GIII2	18466	
6Solid_DIII2	18482	
2Solid_BIII3	18518	
2Solid_BII2	18548	
6Solid_AI2	18549	
2Solid_CII3	18553	
6Solid_DIII1	18644	
6Liquid_GIII2	18656	
6Solid_AIII1	18670	
6Solid_CII3	18695	
6Liquid_EII2	18709	
6Liquid_FII3	18731	
2Solid_DIII3	18763	
6Solid_FII2	18843	
4Liquid_AII2	18881	
6Liquid_FIII1	18954	
6Liquid_AII2	18964	
6Liquid_DI1	18968	
6Liquid_CIII3	18999	
2Solid_FIII3	19015	
2Solid_AIII2	19026	
6Solid_AIII3	19033	
2Solid_HII3	19117	
6Solid_FII3	19136	

continued/-

Table S1. Rumen samples included and excluded from the analysis

continued from previous page

Sample ID	Read count	Status
6Solid_DI3	19191	Included
2Solid_AII3	19237	
6Solid_HI3	19250	
6Solid_FI1	19287	
6Liquid_BIII2	19310	
6Solid_HIII2	19462	
2Solid_DI3	19684	
6Solid_GII3	19743	
2Solid_FII1	19777	
2Solid_CII1	19848	
2Solid_FIII2	19863	
2Solid_GII3	19885	
4Liquid_BI3	19899	
6Solid_DI1	19916	
2Solid_AII2	19942	
2Solid_EIII3	19965	
2Solid_DII3	20035	
6Solid_CIII1	20059	
6Solid_DI2	20131	
6Solid_HI2	20181	
2Solid_AI3	20304	
2Solid_GIII1	20468	
6Solid_HI1	20476	
6Solid_GI3	20674	
6Liquid_CIII1	20725	
2Solid_FII2	20737	
6Liquid_HI2	20871	
6Solid_EI1	21239	
6Solid_GI2	21407	
6Solid_FIII2	21557	
6Liquid_FII1	21829	
6Solid_DII3	22056	
6Liquid_CIII2	22204	
6Solid_FIII1	22429	
6Solid_GII2	22572	
6Solid_BIII3	22619	
6Solid_BII2	22917	
6Solid_GIII3	23148	
6Liquid_FII2	23570	
6Solid_BII3	23708	
6Liquid_AI3	24411	
6Solid_EI2	24823	
6Solid_EI3	25283	
6Solid_GIII2	28823	
2Solid_AI1	14151	
2Liquid_BIII2	11746	

Table S2. Core microbiome - OTUs present with 95% Prevalence

OTUs present in 95% of all the samples.

Phylum level composition

Phylum	MaxQ-1			KY31			MaxQ-2		
	Liquid	Solid	Number of ASVs	Liquid	Solid	Number of ASVs	Liquid	Solid	Number of ASVs
Bacteroidetes	41.49	41.79	9	38.75	46.44	9	36.47	35.34	9
Fibrobacteres	11.71	12.11	2	10.81	9.19	2	9.38	10.92	2
Firmicutes	41.07	41.47	10	43.84	40.38	10	46.63	49.11	10
Kiritimatiellaeota	0.74	0.69	1	1.16	0.87	1	1.20	0.81	1
Lentisphaerae	1.99	1.01	1	3.16	0.75	1	2.91	0.94	1
Patescibacteria	2.04	2.38	1	1.64	2.14	1	2.74	2.42	1
Verrucomicrobia	0.95	0.54	1	0.64	0.23	1	0.67	0.46	1

Family level composition

Phylum	Family	MaxQ-1			KY31			MaxQ-2		
		Liquid	Solid	Number of ASVs	Liquid	Solid	Number of ASVs	Liquid	Solid	Number of ASVs
Patescibacteria	uncultured rumen bacterium	2.04	2.38	1	1.64	2.14	1	2.74	2.42	1
Bacteroidetes	*Bacteroidales RF16 group*	9.74	1.44	1	4.22	0.85	1	9.41	1.26	1
	Prevotellaceae	24.51	22.43	5	21.17	18.55	5	18.78	17.68	5
	Rikenellaceae	7.23	17.91	3	13.36	27.04	3	8.28	16.40	3
Lentisphaerae	*vadinBE97*	1.99	1.01	1	3.16	0.75	1	2.91	0.94	1
Fibrobacteres	*Fibrobacteraceae*	11.71	12.11	2	10.81	9.19	2	9.38	10.92	2
Verrucomicrobia	*Pedosphaeraceae*	0.95	0.54	1	0.64	0.23	1	0.67	0.46	1
Kiritimatiellaeota	uncultured rumen bacterium	0.74	0.69	1	1.16	0.87	1	1.20	0.81	1
Firmicutes	*Acidaminococcaceae*	4.63	2.59	1	2.77	1.70	1	3.61	2.61	1
	Christensenellaceae	1.38	1.41	1	1.41	1.20	1	1.66	1.71	1
	Lachnospiraceae	10.35	13.06	4	8.55	12.51	4	8.87	13.74	4
	Ruminococcaceae	13.41	12.37	3	17.24	13.92	3	17.25	15.61	3
	Unclassified	11.30	12.03	1	13.87	11.05	1	15.23	15.45	1

continued/-

Table S2. Core microbiome - OTUs present with 95% Prevalence

OTUs present in 95% of all the samples.

Genus level composition

continued from previous page

Phylum	Genus	MaxQ-1			KY31			MaxQ-2		
		Liquid	Solid	Number of ASVs	Liquid	Solid	Number of ASVs	Liquid	Solid	Number of ASVs
Patescibacteria	*Absconditabacteriales (SR1)*	2.04	2.38	1	1.64	2.14	1	2.74	2.42	1
Bacteroidetes	***Bacteroidales RF16***	9.74	1.44	1	4.22	0.85	1	9.41	1.26	1
	Prevotellaceae UCG-001	2.14	2.76	2	2.87	2.25	2	2.18	2.35	2
	Prevotella 1	22.37	19.67	3	18.30	16.30	3	16.60	15.34	3
	RikenellaceaeSP3-e08	0.77	1.50	1	1.21	1.32	1	1.16	1.40	1
	Rikenellaceae RC9	6.47	16.41	2	12.15	25.71	2	7.12	15.00	2
Lentisphaerae	***Victivallales vadinBE97***	1.99	1.01	1	3.16	0.75	1	2.91	0.94	1
Fibrobacteres	***Fibrobacter***	11.71	12.11	2	10.81	9.19	2	9.38	10.92	2
Verrucomicrobia	*Pedosphaeraceae*	0.95	0.54	1	0.64	0.23	1	0.67	0.46	1
Kiritimatiellaeota	*KiritimatiellaeWCHB1-41*	0.74	0.69	1	1.16	0.87	1	1.20	0.81	1
Firmicutes	***Succiniclasticum***	4.63	2.59	1	2.77	1.70	1	3.61	2.61	1
	Christensenellaceae R-7	1.38	1.41	1	1.41	1.20	1	1.66	1.71	1
	Pseudobutyrivibrio	3.16	5.27	1	1.90	4.78	1	1.95	4.43	1
	Lachnospiraceae XPB1014	1.70	1.66	1	2.48	2.08	1	3.04	3.36	1
	Butyrivibrio 2	5.49	6.13	2	4.18	5.65	2	3.88	5.96	2
	Saccharofermentans	3.06	5.20	1	3.46	4.33	1	3.32	5.63	1
	Ruminococcaceae NK4A214	10.36	7.17	2	13.78	9.60	2	13.93	9.98	2
	Unclassified Clostridiales	11.30	12.03	1	13.87	11.05	1	15.23	15.45	1

Genera shown in bold have been discussed in the Results and Discussion.

Table S3. Microbiota shift due to grazing transitions

From MaxQ-1 to KY-31

Lowest taxonomic annotation	Relative Abundance	Average Log2 Fold	OTU count
[Anaerorhabdus] furcosa group	0.048189335	1.969577811	1
[Eubacterium] coprostanoligenes group	0.025536099	3.908662791	1
[Eubacterium] ruminantium group	0.069857322	3.159374494	1
[Eubacterium] ventriosum group	0.037921087	4.304509321	1
Acetitomaculum	0.635293569	2.583369685	2
Anaeroplasma	0.272889286	3.32800137	2
Candidatus Endomicrobium	0.294227757	1.422238857	1
Christensenellaceae R-7 group	0.036834767	1.4802637	1
Coprococcus 1	0.069839796	3.022458971	1
Desulfovibrio	0.094531188	2.658513821	1
Erysipelotrichaceae UCG-009	0.308074513	2.643876322	2
Kiritimatiellae WCHB1-41	0.354875875	1.295996345	1
Lachnospiraceae FCS020 group	0.040797913	2.520554391	1
Lachnospiraceae NK3A20 group	1.946298265	2.973764949	4
Lachnospiraceae NK4A136 group	0.171388271	0.611286272	1
Lachnospiraceae XPB1014 group	1.100138325	3.139407524	2
Methanobrevibacter	0.089443331	3.20898017	1
Mycoplasma	0.079950685	3.041932772	1
Paraprevotella	0.265396825	3.31782513	1
Prevotella 1	7.936418991	3.886763024	16
Prevotellaceae UCG-001	1.686410667	1.589199511	7
Prevotellaceae UCG-003	0.748453883	0.881379302	5
Rikenellaceae RC9 gut group	7.82315993	1.244754355	7
Ruminococcaceae NK4A214 group	4.977310548	2.044980344	6
Ruminococcaceae UCG-005	0.056104642	2.189201463	1
Ruminococcaceae UCG-014	0.78624257	1.418549215	4
Ruminococcus 1	0.530237724	2.100611711	1
Saccharofermentans	0.285192174	0.907426675	1
Solobacterium	0.081813743	0.976159509	1
uncultured Bacteroidales	0.603476189	1.019628723	2
uncultured Bacteroidales F082	1.779772952	1.238978735	5
uncultured Bacteroidales p-251-o5	3.442720648	1.302412896	3
uncultured Bacteroidales UCG-001	0.128283387	1.426073909	1
uncultured Muribaculaceae	0.694011405	1.766822037	7
unidentified Mollicutes RF39	0.999410418	1.929249341	4
Victivallales vadinBE97	0.767777781	2.324389097	2

Positive Log2 fold values indicates enriched microorganisms in KY-31 compared to MaxQ-1

continued/-

Table S3. Microbiota shift due to grazing transitions

From KY-31 to MaxQ-2

continued from p

Lowest taxonomic annotation	Relative Abundance	Average Log2 Fold	OTU count
Bacillales	0.074037044	1.767603311	1
Bacteroidia	0.056785013	4.111301438	1
Christensenellaceae R-7 group	2.585204381	5.571572215	7
Clostridiales	5.711511876	0.332689536	1
Deltaproteobacteria	0.019755231	2.527047387	1
Ethanoligenens	0.052638507	2.185036404	1
Lachnoclostridium 10	0.959286425	3.70332786	5
Lachnospiraceae	0.169867629	3.8254107	2
Lachnospiraceae AC2044 group	0.699910857	0.847845491	1
Lachnospiraceae NK4A136 group	0.040126262	4.40731954	1
Lachnospiraceae XPB1014 group	1.18848974	0.547366231	1
Mollicutes RF39	0.019883714	3.279033918	1
p-1088-a5 gut group	0.046561667	2.122269862	1
Prevotella 1	5.761149446	3.263028843	14
Prevotellaceae	0.216538517	5.417926511	2
Prevotellaceae Ga6A1 group	0.158718911	2.023164847	1
Prevotellaceae UCG-001	0.444695124	4.586064045	4
Prevotellaceae UCG-003	0.244975305	4.449635955	2
Rikenellaceae RC9 gut group	0.239438973	3.111657552	1
Ruminiclostridium 1	0.05767732	4.425959074	1
Ruminiclostridium 6	0.026874544	3.561490147	1
Ruminococcaceae UCG-005	0.033835554	4.137825367	1
Ruminococcaceae UCG-013	0.029940715	3.459706518	1
Ruminococcaceae UCG-014	0.104722657	4.654701499	2
Ruminococcus 1	0.430803015	3.373168583	3
Saccharofermentans	1.756200819	1.349327701	2
SP3-e08	0.212064248	2.303011369	1
Succiniclasticum	1.15558381	0.514885672	1
Treponema 2	0.512189306	2.807594236	4
Tyzzerella 3	0.050008274	1.864055365	1
uncultured Absconditabacteriales (SR1)	1.045600781	1.8490638	3
uncultured Bacteroidales BS11 gut group	1.416026204	1.547454869	1
uncultured Bacteroidales F082	1.441122611	5.036088881	1
uncultured Bacteroidales p-251-o5	0.078355664	5.139120674	1
uncultured Bacteroidales RF16 group	1.936103155	1.026716123	1
uncultured Deltaproteobacteria PB19	0.065947576	1.375861454	1
uncultured Kiritimatiellae WCHB1-41	0.343209063	1.980366903	3
uncultured Muribaculaceae	0.376057735	2.573549919	2
uncultured Oligoflexales 0319-6G20	0.532954513	1.181928716	1
uncultured Rhodospirillales	0.041547182	4.276565515	1
uncultured Ruminococcaceae	0.024821249	3.150730485	1
Veillonellaceae UCG-001	0.20388016	0.681453346	1

Positive Log2 fold values indicates enriched microorganisms in MaxQ-2 compared to KY-31

continued/-

Table S3. Microbiota shift due to grazing transitions

continued from previous page

Between MaxQ-1 and MaxQ-2

Lowest taxonomic annotation	AverageLog2Fold	Group	OTUcount
[Eubacterium] coprostanoligenes	3.91	maxq2	3 (4)
[Eubacterium] hallii	2.00	maxq2	
[Eubacterium] ruminantium	2.47	maxq2	
[Eubacterium] xylanophilum	1.05	maxq2	
[Ruminococcus] gauvreauii	4.13	maxq2	
Acetitomaculum	6.14	maxq2	1 (1)
Allorhizobium	3.15	maxq2	1 (2)
Anaeroplasma	2.54	maxq2	4 (8)
Candidatus Saccharimonas	2.95	maxq2	
Christensenellaceae R-7	4.28	maxq2	2 (2)
Coprococcus 1	1.94	maxq2	
Desulfovibrio	1.78	maxq2	
Erysipelotrichaceae UCG-004	3.52	maxq2	6 (9)
Erysipelotrichaceae UCG-009	3.63	maxq2	1 (2)
Ethanoligenens	1.61	maxq2	1 (1)
Flexilinea	3.25	maxq2	
Lachnospiraceae ND3007	3.48	maxq2	2 (2)
Lachnospiraceae NK3A20	2.72	maxq2	1 (3)
Methanobrevibacter	1.65	maxq2	1 (1)
Mogibacterium	0.80	maxq2	
Mycoplasma	2.09	maxq2	1 (1)
p-1088-a5	2.04	maxq2	1 (1)
Prevotella 1	2.42	maxq2	16 (26)
Prevotellaceae UCG-001	2.16	maxq2	
Prevotellaceae UCG-003	3.48	maxq2	12 (16)
Pseudobutyrivibrio	5.30	maxq2	1 (1)
Rikenellaceae RC9	3.64	maxq2	3 (7)
Ruminiclostridium 1	4.45	maxq2	1 (1)
Ruminiclostridium 9	4.30	maxq2	
Ruminococcaceae NK4A214	0.69	maxq2	2 (2)
Ruminococcaceae UCG-010	3.02	maxq2	3 (6)
Ruminococcaceae UCG-013	4.07	maxq2	2 (2)
Ruminococcus 1	3.72	maxq2	
Ruminococcus 2	4.70	maxq2	1 (1)
Streptococcus	2.82	maxq2	1 (1)
Tyzzerella 3	1.69	maxq2	1 (1)
uncultured Bacteria	0.93	maxq2	
uncultured Bacteroidales BS11	3.05	maxq2	4 (5)
uncultured Bacteroidales F082	4.49	maxq2	5 (6)
uncultured Bacteroidales p-251-o5	3.06	maxq2	1 (1)
uncultured Bacteroidales RF16	2.99	maxq2	3 (5)
uncultured Clostridiales vadinBB60	4.01	maxq2	2 (2)
uncultured Izimaplasmatales	3.02	maxq2	
uncultured Kiritimatiellae WCHB1-41	3.53	maxq2	12 (18)
uncultured Lachnospiraceae	3.81	maxq2	4 (5)
uncultured Methanomethylophilaceae	3.52	maxq2	1 (3)
uncultured Mollicutes RF39	3.28	maxq2	
uncultured Oligosphaeraceae	2.92	maxq2	
uncultured Pedosphaeraceae	0.64	maxq2	
uncultured Ruminococcaceae	3.12	maxq2	1 (2)
uncultured Veillonellaceae	4.07	maxq2	1 (1)
uncultured Victivallaceae	2.32	maxq2	
uncultured Victivallales vadinBE97	2.83	maxq2	3 (3)

continued/-

Table S3. Microbiota shift due to grazing transitions

Between MaxQ-1 and MaxQ-2

continued from previous page

Lowest taxonomic annotation	AverageLog2Fold	Group	OTUcount
[Eubacterium] ruminantium	-1.68	maxq1	
Azoarcus	-3.14	maxq1	
Blautia	-2.04	maxq1	1 (2)
Butyrivibrio 2	-1.58	maxq1	
Christensenellaceae R-7	-3.66	maxq1	2 (4)
Fibrobacter	-2.09	maxq1	2 (3)
Flexilinea	-2.91	maxq1	
Lachnobacterium	-4.55	maxq1	
Lachnoclostridium 10	-1.92	maxq1	
Lachnospiraceae AC2044	-1.65	maxq1	1 (4)
Lachnospiraceae FCS020	-3.14	maxq1	
Lachnospiraceae ND3007	-1.01	maxq1	
Lachnospiraceae NK3A20	-1.01	maxq1	
Lachnospiraceae NK4A136	-2.71	maxq1	
Lachnospiraceae UCG-008	-0.65	maxq1	
Mailhella	-1.18	maxq1	1 (1)
Methanobrevibacter	-4.43	maxq1	
Oribacterium	-3.00	maxq1	
Papillibacter	-1.99	maxq1	
Paraprevotella	-1.94	maxq1	1 (1)
Prevotella 1	-2.40	maxq1	19 (34)
Prevotellaceae NK3B31	-2.79	maxq1	9 (12)
Prevotellaceae UCG-001	-2.14	maxq1	1 (8)
Prevotellaceae UCG-003	-1.26	maxq1	
Prevotellaceae UCG-004	-1.90	maxq1	1 (2)
Pseudobutyrivibrio	-1.48	maxq1	1 (1)
Pyramidobacter	-2.60	maxq1	1 (1)
Rikenellaceae RC9	-2.16	maxq1	7 (10)
Rikenellaceae SP3-e08	-0.44	maxq1	1 (1)
Ruminiclostridium 1	-1.81	maxq1	1 (1)
Ruminococcaceae NK4A214	-5.14	maxq1	
Ruminococcaceae UCG-010	-2.74	maxq1	1 (3)
Ruminococcaceae UCG-014	-2.47	maxq1	
Ruminococcus 1	-2.95	maxq1	1 (1)
Saccharofermentans	-2.01	maxq1	3 (4)
Shuttleworthia	-2.33	maxq1	
SP3-e08	-1.82	maxq1	
Succiniclasticum	-1.01	maxq1	
Succinivibrio	-1.54	maxq1	
Treponema 2	-2.52	maxq1	11 (14)
uncultured Bacteroidales	-1.23	maxq1	3 (3)
uncultured Bacteroidales BS11	-1.55	maxq1	1 (1)
uncultured Bacteroidales F082	-1.31	maxq1	
uncultured Bacteroidales p-251-o5	-2.49	maxq1	6 (7)
uncultured Bacteroidetes BD2-2	-1.12	maxq1	
uncultured Bacteroidia	-3.26	maxq1	1 (2)
uncultured Clostridiales	-1.93	maxq1	
uncultured Gastranaerophilales	-2.42	maxq1	
uncultured Lachnospiraceae	-3.04	maxq1	3 (4)
uncultured Methanomethylophilaceae	-3.85	maxq1	1 (2)
uncultured Mollicutes RF39	-1.54	maxq1	
uncultured Muribaculaceae	-3.43	maxq1	2 (3)
uncultured Prevotellaceae	-3.55	maxq1	1 (2)
uncultured Ruminococcaceae	-3.14	maxq1	

Table S4. Microbiota shift in specific rumen fraction (solid and liquid) due to grazing transitions

Grazing transition: MaxQ-1 to KY31 (fold change in KY31 with respect to MaxQ-1)

In Solid Fraction (sessile population)

Lowest taxonomic annotation		OTU count	Average Log2 Fold Change
Bacteroidales F082	KY31	1	1.395395968
Fibrobacter	MaxQ-1	2	-2.284049733
Lachnospiraceae AC2044	MaxQ-1	1	-0.826241407
Lachnospiraceae FCS020	KY31	1	3.139407524
Paraprevotella	KY31	1	1.418549215
Prevotella 1	KY31	5	1.535175237
Prevotellaceae UCG-001	KY31	2	1.607969976
Rikenellaceae RC9	KY31	3	1.474034184
Saccharofermentans	MaxQ-1	1	-1.177387036
uncultured *Bacteroidales*	KY31	1	1.783220396
uncultured *Bacteroidales p-251-o5*	MaxQ-1	1	-1.644329435
uncultured *Bacteroidales p-251-o5*	KY31	2	1.623981056
uncultured *Kiritimatiellae WCHB1-41*	MaxQ-1	1	-0.512205654
uncultured *Muribaculaceae*	KY31	1	2.287184456
uncultured *Spirochaetes MVP-15*	MaxQ-1	1	-1.704765761

In Liquid Fraction (planktonic population)

Lowest taxonomic annotation		OTU count	Average Log2 Fold Change
Acetitomaculum	KY31	1	3.32800137
Anaeroplasma	KY31	1	1.4802637
Coprococcus 1	KY31	1	2.643876322
Desulfovibrio	KY31	1	1.295996345
Erysipelotrichaceae UCG-009	KY31	1	2.067343832
Lachnospiraceae NK3A20	KY31	2	3.464347898
Mycoplasma	KY31	1	2.189201463
Prevotella 1	MaxQ-1	3	-2.928526409
Prevotella 1	KY31	3	1.213737385
Prevotellaceae UCG-001	KY31	2	3.046436534
Prevotellaceae UCG-003	MaxQ-1	1	-4.152963353
Prevotellaceae UCG-003	KY31	1	2.90543508
Rikenellaceae RC9	MaxQ-1	2	-2.666591878
Ruminococcaceae NK4A214	KY31	1	0.458738105
Ruminococcaceae UCG-013	MaxQ-1	1	-4.071140948
Succiniclasticum	MaxQ-1	1	-0.540357438
uncultured Bacteroidales BS11	MaxQ-1	1	-1.52934446
uncultured Bacteroidales RF16	MaxQ-1	1	-0.920903743
uncultured *Pedosphaeraceae*	MaxQ-1	1	-0.654350079
Victivallales vadinBE97	KY31	2	1.149146792

continued/-

Table S4. Microbiota shift in specific rumen fraction (solid and liquid) due to grazing transitions

Grazing transition: KY31 and MaxQ-2 (fold change in MaxQ-2 with respect to KY31)

In Solid Fraction (sessile population)

continued from previous page

Lowest taxonomic annotation		OTU count	Average Log2 Fold Change
Paraprevotella	KY31	1	1.593293252
Prevotella 1	KY31	6	1.646635416
Prevotellaceae NK3B31	KY31	1	1.450810084
Rikenellaceae RC9	KY31	2	1.259296379
uncultured *Bacteroidales*	KY31	2	1.023361584
uncultured *Bacteroidales p-251-o5*	KY31	3	1.508989828
Lachnoclostridium 10	MaxQ-2	2	-2.198358303
Lachnospiraceae AC2044	MaxQ-2	1	-0.847845491
Christensenellaceae R-7	MaxQ-2	1	-0.610066474
Saccharofermentans	MaxQ-2	1	-0.281319093
Treponema 2	MaxQ-2	2	-2.111047884

In Liquid Fraction (planktonic population)

Lowest taxonomic annotation		OTU count	Average Log2 Fold Change
Anaeroplasma	KY31	2	1.720066419
Desulfovibrio	KY31	1	1.377557133
Erysipelotrichaceae UCG-004	KY31	1	2.38592285
Ethanoligenens	MaxQ-2	1	-2.185036404
Mycoplasma	KY31	1	1.941421353
p-1088-a5	MaxQ-2	1	-2.122269862
Prevotella 1	KY31	3	0.534951799
Prevotella 1	MaxQ-2	3	-4.321944124
Prevotellaceae UCG-001	KY31	1	0.798318273
Prevotellaceae UCG-003	KY31	1	1.561342875
Prevotellaceae UCG-003	MaxQ-2	1	-4.693749775
Rikenellaceae RC9	MaxQ-2	1	-4.088374447
Ruminococcaceae UCG-013	MaxQ-2	1	-3.459706518
Tyzzerella 3	MaxQ-2	1	-1.864055365
uncultured Bacteroidales BS11	MaxQ-2	1	-1.547454869
uncultured *Bacteroidales p-251-o5*	MaxQ-2	1	-5.139120674
uncultured Bacteroidales RF16	MaxQ-2	1	-1.026716123
uncultured *Kiritimatiellae WCHB1-41*	KY31	1	3.514795388
uncultured *Kiritimatiellae WCHB1-41*	MaxQ-2	1	-2.943817078
uncultured *Ruminococcaceae*	MaxQ-2	1	-3.150730485

continued/-

Table S4. Microbiota shift in specific rumen fraction (solid and liquid) due to grazing transitions

Grazing transition: MaxQ-1 and MaxQ-2 (fold change in MaxQ-1 with respect to MaxQ-2)

In Solid Fraction (sessile population)

continued from previous page

Lowest taxonomic annotation		OTU count	Average Log2 Fold Change
Anaeroplasma	MaxQ-2	2	-1.925053167
Prevotella 1	MaxQ-2	2	-2.031622133
uncultured *Bacteroidales BS11*	MaxQ-2	1	-1.402442612
Butyrivibrio 2	MaxQ-1	1	1.461765124
Prevotella 1	MaxQ-1	2	1.578030962

In Liquid Fraction (planktonic population)

Lowest taxonomic annotation		OTU count	Average Log2 Fold Change
Eubacterium hallii	MaxQ-2	1	-2.002055032
Eubacterium ruminantium	MaxQ-1	1	1.745958773
Eubacterium xylanophilum	MaxQ-2	1	-1.046047444
Christensenellaceae R-7	MaxQ-2	1	-4.062037109
Christensenellaceae R-7	MaxQ-1	2	2.631853784
Erysipelotrichaceae UCG-009	MaxQ-2	1	-3.666810214
Lachnoclostridium 10	MaxQ-1	1	1.922176696
Lachnospiraceae AC2044	MaxQ-1	1	2.120457866
Lachnospiraceae ND3007	MaxQ-1	1	1.009264316
Lachnospiraceae NK3A20	MaxQ-2	1	-1.384930407
Methanobrevibacter	MaxQ-2	1	-1.653843139
Mollicutes RF39	MaxQ-2	1	-2.425710928
p-1088-a5	MaxQ-2	1	-2.044989428
Prevotella 1	MaxQ-2	2	-1.266701958
Prevotella 1	MaxQ-1	1	1.127608252
Prevotellaceae UCG-001	MaxQ-2	2	-2.024443779
Prevotellaceae UCG-001	MaxQ-1	2	2.411783425
Prevotellaceae UCG-003	MaxQ-2	4	-3.316968155
Prevotellaceae UCG-004	MaxQ-1	1	2.628645529
Rikenellaceae RC9	MaxQ-1	2	0.992077828
Ruminococcaceae NK4A214	MaxQ-2	2	-0.686516815
Ruminococcaceae UCG-010	MaxQ-2	1	-2.926997757
Ruminococcaceae UCG-010	MaxQ-1	1	2.203666677
Shuttleworthia	MaxQ-1	1	2.327207749
Treponema 2	MaxQ-1	1	2.052530647
Tyzzerella 3	MaxQ-2	1	-1.689394097
uncultured *Bacteroidales*	MaxQ-1	1	0.993517475
uncultured *Bacteroidales BS11*	MaxQ-2	1	-2.33526434
uncultured *Bacteroidales RF16*	MaxQ-2	1	-2.145434433
uncultured *Kiritimatiellae WCHB1-41*	MaxQ-2	1	-0.256792691
Victivallales vadinBE97	MaxQ-2	2	-2.327524334

Table S5. Differentially abundant prokaryotic OTUs in the rumen of susceptible steers with respect to that of tolerant steers

Susceptibility to fescue toxicosis as defined by DRD2 gene polymorphism and described in the Materials and Methods

MaxQ-1 grazing

Average Log2 Fold Change	Lowest taxonomic annotation		OTU count
-5.319410941	*Eubacterium coprostanoligenes*	tolerant	1
-5.722359219	*Anaeroplasma*	tolerant	1
0.832309468	*Anaeroplasma*	susceptible	1
1.151262108	*Anaerovorax*	susceptible	1
3.567157398	*Bacillales*	susceptible	1
4.476520578	*Butyrivibrio 2*	susceptible	2
-3.526663692	*Campylobacter*	tolerant	1
1.062271501	*Christensenellaceae R-7*	susceptible	1
-4.530663527	*Fibrobacter*	tolerant	1
0.842744797	*Fibrobacter*	susceptible	1
-5.809402984	*Gastranaerophilales*	tolerant	1
-1.241349762	*Lachnospiraceae AC2044*	tolerant	1
6.00629164	*Lachnospiraceae AC2044*	susceptible	1
5.386865921	*Lachnospiraceae NK4A136*	susceptible	1
-4.705606283	*Mollicutes RF39*	tolerant	3
4.972127855	*Mollicutes RF39*	susceptible	6
-4.164266944	*Pantoea*	tolerant	1
-2.288636892	*Pirellulaceae CPla-4 termite*	tolerant	1
-3.740844693	*Prevotella 1*	tolerant	4
4.309163957	*Prevotella 1*	susceptible	8
-4.075596129	*Prevotellaceae UCG-001*	tolerant	2
3.238266115	*Prevotellaceae UCG-001*	susceptible	1
-4.282915069	*Prevotellaceae UCG-003*	tolerant	3
-3.944741019	*Prevotellaceae UCG-004*	tolerant	2
5.16629755	*Prevotellaceae UCG-004*	susceptible	1
0.585032844	*Pseudobutyrivibrio*	susceptible	1
-8.196955628	*Rikenellaceae RC9*	tolerant	6
4.325381459	*Rikenellaceae RC9*	susceptible	6
0.672891276	*Rikenellaceae SP3-e08*	susceptible	1
-2.422780228	*Rikenellaceae U29-B03*	tolerant	1
3.787731698	*Ruminococcaceae UCG-005*	susceptible	1
-4.14324446	*Ruminococcaceae UCG-014*	tolerant	1
6.418746205	*Ruminococcaceae UCG-014*	susceptible	1
-5.953087395	*Saccharofermentans*	tolerant	1
4.748716977	*Shuttleworthia*	susceptible	1
-0.830504912	*Sutterella*	tolerant	1
-4.010747131	*Treponema 2*	tolerant	1
5.511151573	*Treponema 2*	susceptible	1
-3.608279027	uncultured *Absconditabacteriales (SR1)*	tolerant	2
-5.158835735	uncultured *Bacteroidales*	tolerant	2
5.676635268	uncultured *Bacteroidales*	susceptible	1
-7.360496247	uncultured *Bacteroidales BS11*	tolerant	1
-6.608478919	uncultured *Bacteroidales F082*	tolerant	2
5.263466895	uncultured *Bacteroidales F082*	susceptible	3
-6.171889559	uncultured *Bacteroidales UCG-001*	tolerant	1
5.784624154	uncultured *Bacteroidetes BD2-2*	susceptible	1
-3.832248702	uncultured *Marinilabiliaceae*	tolerant	1

continued/-

Table S5. Differentially abundant prokaryotic OTUs in the rumen of susceptible steers with respect to that of tolerant steers

MaxQ-1 grazing (continued)

continued from previous page

Average Log2 Fold Change	Lowest taxonomic annotation		OTU count
1.150121749	uncultured *MollicutesNED5E9*	susceptible	1
-4.924575892	uncultured *Muribaculaceae*	tolerant	6
4.625161989	uncultured *Muribaculaceae*	susceptible	6
3.8410405	uncultured *Paenibacillaceae*	susceptible	1
-5.707215708	uncultured *Prevotellaceae*	tolerant	1
6.358953159	uncultured *Prevotellaceae*	susceptible	2
-4.477827525	uncultured *Rhodospirillales*	tolerant	2
-4.574139713	*Verrucomicrobiae LD1-PB3*	tolerant	1
-0.733917875	*Victivallales vadinBE97*	tolerant	1

KY31 grazing

Average Log2FoldChange	Lowest taxonomic annotation		OTU count
5.912764472	*Christensenellaceae R-7*	susceptible	1
-3.688847825	*Kiritimatiellae WCHB1-41*	tolerant	1
4.409961449	*Kiritimatiellae WCHB1-41*	susceptible	1
-2.940428333	*Mollicutes RF39*	tolerant	1
6.988374844	*Mollicutes RF39*	susceptible	1
2.106036202	*Mycoplasma*	susceptible	1
-1.026116129	*Prevotella 1*	tolerant	2
3.178496865	*Prevotella 1*	susceptible	6
-1.469844434	*Prevotellaceae UCG-001*	tolerant	1
-6.571617045	*Prevotellaceae UCG-003*	tolerant	1
3.757064529	*Prevotellaceae UCG-003*	susceptible	2
6.067539091	*Rikenellaceae RC9*	susceptible	1
2.304615329	*Ruminococcaceae NK4A214*	susceptible	2
0.920487459	*Ruminococcaceae UCG-005*	susceptible	1
6.423748029	*Ruminococcaceae UCG-014*	susceptible	1
-5.157956789	*Saccharofermentans*	tolerant	3
-4.105186201	uncultured *Bacteroidales*	tolerant	1
1.881941218	uncultured *Bacteroidales*	susceptible	1
-9.373237203	uncultured *Bacteroidales F082*	tolerant	4
14.17952821	uncultured *Bacteroidales F082*	susceptible	2
-4.804208734	uncultured *Bacteroidales p-251-o5*	tolerant	1
4.086936067	uncultured *Bacteroidales PeH15*	susceptible	1
-7.061536006	uncultured *Bacteroidales UCG-001*	tolerant	1
-3.704739584	uncultured *Bacteroidia*	tolerant	1
-3.063632277	uncultured *Deltaproteobacteria PB19*	tolerant	1
-4.778985173	uncultured *Muribaculaceae*	tolerant	2
4.129862483	uncultured *Muribaculaceae*	susceptible	4

continued/-

Table S5. Differentially abundant prokaryotic OTUs in the rumen of susceptible steers with respect to that of tolerant steers

MaxQ-2 grazing

continued from previous page

Average Log2 Fold Change	Lowest taxonomic annotation		OTU count
5.042009848	*Christensenellaceae R-7*	susceptible	1
-5.919861942	*Fibrobacter*	tolerant	1
-3.056389422	*Mollicutes RF39*	tolerant	2
3.143769193	*Pirellulaceae CPla-4 termite*	susceptible	1
-4.437930405	*Prevotella 1*	tolerant	2
5.111194237	*Prevotella 1*	susceptible	5
1.160480533	*Prevotellaceae UCG-001*	susceptible	1
-4.430838196	*Prevotellaceae UCG-004*	tolerant	1
-2.686191417	*Rikenellaceae RC9*	tolerant	2
4.006571774	*Rikenellaceae RC9*	susceptible	2
-2.528864413	*Rikenellaceae U29-B03*	tolerant	1
-3.001655687	*Ruminococcaceae UCG-014*	tolerant	3
-6.320707641	*Ruminococcus 1*	tolerant	1
-4.174349651	*Spirochaetaceae M2PT2-76 termite*	tolerant	1
-4.616737495	*Treponema 2*	tolerant	1
-5.5860467	uncultured *Bacteria*	tolerant	1
-3.6919519	uncultured *Bacteroidales BS11*	tolerant	2
-3.759287193	uncultured *Bacteroidales F082*	tolerant	4
5.112252121	uncultured *Bacteroidales F082*	susceptible	2
-2.372037979	uncultured *Bacteroidales p-251-o5*	tolerant	1
-3.538227106	uncultured *Bacteroidales RF16*	tolerant	1
-6.335319325	uncultured *Gastranaerophilales*	tolerant	1
-2.387164099	uncultured *Marinilabiliaceae*	tolerant	1
-1.204621307	uncultured *Pedosphaeraceae*	tolerant	1
-4.774274489	Uncultured *Prevotellaceae*	tolerant	1
-6.279496538	uncultured *Rhodospirillales*	tolerant	1

Table S6. Phylum-level microbiota comparison between steers grazing two tall fescue cultivars and fed other diets

Phyla / Diet Type	Forage (43)	Mixed Forage (43)	High Grain (43)	Pure Alfalfa - bloated cows (45)				Bermuda Grass (47)	Sunn Hemp forage (47)
	Rumen Overall	Rumen Overall	Rumen Overall	Rumen Overall	Rumen Solid	Rumen Liquid	Fecal	Rumen Overall	Rumen Overall
Actinobacteria				0.31%	0.40%	0.21%	0.16%		
Bacteroidetes	25.70%	26.20%	40.30%	55.71%	53 ± 9.9%	61.1 ± 9.9%	28.51 ± 4.4%	34% - 38%	34% - 38%
Chloroflexi				<1%	0%	0	0%		
Cyanobacteria	-	-	1.80%	-	-	-	-		
Elusimicrobia				0.29%	0.23%	0.35%	0.23%		
Epsilonbacteraeota				-	-	-	-		
Euryarchaeota				-	-	-	-	-	-
Fibrobacteres	7.10%	-	-	4.07%	4.90%	3.10%	0.01%		
Firmicutes	55.20%	55.80%	37.00%	32.10%	33.9 ± 8.3%	26.7 ± 6.9%	63.36 ± 4.9%	45%- 48%	45%- 48%
Fusobacteria				<1%	0%	0%	-		
Lentisphaerae				<1%	0%	0%	0%		
Planctomycetes				-	-	-	-		
Proteobacteria	4.70%	8.90%	17.90%	0.84%	0.54 ± 0.32%	0.84 ± 0.42%	0.83 ± 0.3%	-	-
Spirochaetes	2.80%	-	-	1.60%	1.90%	1.21%	0.90 ± 0.87%		
Synergistetes				0.19%	0.22%	0.15%	0		
Tenericutes				0.63%	0.52%	0.73%	0.34 ± 0.18%		
Verrucomicrobia				<1%	0%	0%	0		

continued/-

References are listed in the main manuscript.

Table S6. Phylum-level microbiota comparison between steers grazing two tall fescue cultivars and fed other diets

continued from previous page

Phyla / Diet Type	Perennial Ryegrass (46)			White Clover (46)			Ryegrass and clover pasture (48)	Hard-red winter Wheat Pasture (49)	Green Roughage (51)	Green roughage and concentrate (51)
	Rumen Overall	Rumen Solid	Rumen Liquid	Rumen Overall	Rumen Solid	Rumen Liquid	Rumen Overall	Rumen Overall	Rumen Overall	Rumen Overall
Actinobacteria	4.42%	3.72%	5.12%	3.00%	3.10%	2.90%	0.34%			
Bacteroidetes	44.32%	27.90%	61.50%	39.34%	25.90%	53.10%	11.80%	59-77%		
Chloroflexi	0.19%	0.25%	0.12%	0.11%	0.20%	0.03%	0.03%			
Cyanobacteria	0.63%	0.19%	1.08%	0.38%	0.23%	0.53%	0.12%			
Elusimicrobia	<1%	0.02%	0.02%	<1%	0.01%	0.01%	0.03%			
Epsilonbacteraeota	-	-	-	-	-	-	-			
Euryarchaeota	0.83%	1.46%	0.19%	1.55%	2.93%	0.17%	-		81.80%	91.60%
Fibrobacteres	5.21%	8.12%	2.29%	1.78%	2.21%	1.36%	2.37%			
Firmicutes	38.62%	49.80%	27.90%	48.79%	59.80%	38.10%	82.10%	20-33%%	1.40%	6.17%
Fusobacteria	-	-	-	-	-	-	-			
Lentisphaerae	<1%	0.07%	0.02%	<1%	0.01%	0.01%	0.03%			
Planctomycetes	<1%	0.03%	0.01%	<1%	0.04%	0%	0.02%			
Proteobacteria	0.36%	0.30%	0.42%	1.00%	0.88%	1.13%	0.05%			56%
Spirochaetes	3.45%	5.91%	0.99%	1.98%	2.85%	1.10%	0.45%	1-8%		
Synergistetes	<1%	0.03%	0%	<1%	0.06%	0.01%	0.04%			
Tenericutes	1.26%	1.66%	0.87%	1.51%	1.57%	1.45%	0.54%			
Verrucomicrobia	0.54%	0.65%	0.43%	0.38%	0.38%	0.39%	0.01%			

continued/-

References are listed in the main manuscript.

Table S6. Phylum-level microbiota comparison between steers grazing two tall fescue cultivars and fed other diets

* average relative abundance ± standard deviation at phyla-level based on tall fescue variety and rumen fraction

continued from previous page

Phyla / Diet Type	Tall Fescue (KY31)					Tall Fescue (MaxQ)			
	Fecal (14)	Fecal (41)	Rumen - Overall (this study*)	Rumen - Solid (this study*)	Rumen -Liquid (this study*)	Fecal (14)	Rumen - Overall (this study*)	Rumen - Solid (this study*)	Rumen -Liquid (this study*)
Actinobacteria		1.1 - 3.7%	0.09% ± 0.14	0.04% ± 0.06	0.14% ± 0.17		0.05% ± 0.07	0.06% ± 0.07	0.05% ± 0.074
Bacteroidetes	27-37%	19.7 - 25.7%	51.32% ± 6.04	54.82% ± 4.95	47.97% ± 5.02	27-37%	50.26% ± 4.37	50.46% ± 5.4	50.07% ± 3.12
Chloroflexi		<1%	0.5% ± 0.32	0.5% ± 0.34	0.51% ± 0.3		0.39% ± 0.22	0.39% ± 0.2	0.39% ± 0.24
Cyanobacteria		<1%	0.5% ± 0.37	0.58% ± 0.38	0.41% ± 0.33		0.90% ± 0.51	0.82% ± 0.22	1% ± 0.68
Elusimicrobia		<1%	0.2% ± 0.24	0.32% ± 0.27	0.23% ± 0.19		0.26% ± 0.16	0.24% ± 0.13	0.27% ± 0.18
Epsilonbacteraeota		<1%	<0.01%	<0.01% ± 0.004	<0.01% ± 0.0007		0.12% ± 0.15	0.09% ± 0.12	0.15% ± 0.17
Euryarchaeota	-	0.80%	0.87% ± 0.36	0.68% ± 0.28	1.04% ± 0.34		0.77% ± 0.34	0.61% ± 0.22	0.92% ± 0.36
Fibrobacteres		<1%	3.24% ± 1.62	3.24% ± 1.84	3.23% ± 1.4		4.73% ± 1.86	5.41% ± 2.12	4.08% ± 1.28
Firmicutes	53-61%	58.7 - 67.9%	33.09% ± 5.75	31.54% ± 4.8	34.59% ± 6.2		30.49% ± 4.20	30.71% ± 3.83	30.26% ± 4.54
Fusobacteria		<1%	<0.01%	<0.01% ± 0.006	<0.01% ± 0.003		<0.01%	<0.01% ± 0.02	0.01% ± 0.044
Lentisphaerae		<1%	0.68% ± 0.5	0.25% ± 0.18	1.1% ± 0.34		0.50% ± 0.32	0.31% ± 0.2	0.67% ± 0.32
Planctomycetes		<1%	0.14% ± 0.13	0.1% ± 0.07	0.18% ± 0.16		0.16% ± 0.22	0.1% ± 0.08	0.21% ± 0.3
Proteobacteria		1.1 - 12.8%	0.93% ± 0.33	0.9% ± 0.31	0.97% ± 0.34		1.62% ± 0.62	1.5 % ± 0.38	1.72% ± 0.76
Spirochaetes		<1%	1.46% ± 0.9	2% ± 0.98	0.93% ± 0.31		1.91% ± 1.35	2.92 % ± 1.25	0.93% ± 0.39
Synergistetes		<1%	0.12% ± 0.07	0.14% ± 0.07	0.09% ± 0.06		0.11% ± 0.07	0.15% ± 0.06	0.06% ± 0.06
Tenericutes		<1%	4.44% ± 1.49	3.36% ± 0.9	5.48% ± 1.16		4.93% ± 1.60	4.26% ± 1.04	5.57% ± 1.77
Verrucomicrobia		<1%	0.18% ± 0.01	0.12% ± 0.06	0.23% ± 0.09		0.33% ± 0.19	0.25% ± 0.13	0.4% ± 0.2

References are listed in the main manuscript.
